# An Improved Method of Medication

**Published:** 1886-09-20

**Authors:** 


### AN IMPROVED METHOD OF MEDICATION.

If the physician of the last century, who made his infusions and decoctions, and pills, in his own private laboratory, could be restored to the practice of his profession at the present day and have at his command the variety of medical preparations, in concentrated and palatable form, that the enterprise and skill of pharmacists have introduced, he must feel as if his occupation were gone, or certainly that portion of it which had to do with the preparation of medicines. The improvements in pharmacy have been so many and so important that we doubt if there are many physicians to-day who have kept pace with them, and who are fully informed regarding the preparation of drugs and methods of administering them acceptably to patients, and yet given equal opportunities and equal medical acquirements that physician who applies in his practice the improved methods pharmacy has provided, and is constantly adding to, reaps the largest share of pecuniary success.

Since the discovery of the subcutaneous method of medication many drugs have been prepared in forms which admit of their being administered hypodermically. The convenience and accuracy of this method must grow to be more generally appreciated, and we doubt not it will in time supersede, in many cases, the exhibition of medicine per os. The improvements in hypodermic syringes and the manufacture of a sufficient variety of tablets to meet the requirements of practice has given a further impetus to the hypodermic method. It will be gratifying to our readers, therefore, to learn that Messrs. Parke, Davis & Co., with their characteristic prompt recognition of the demands of the profession, now offer a very complete line of Hypodermic Tablets, which they supply in small glass tubes, convenient for carrying in the pocket or medicine case, each tube containing 25 tablets. For the convenience of physicians, they have also devised a Pocket-Case, containing an improved hypodermic syringe especially adapted for the use of these tablets and six tubes of tablets. Any formulæ which physicians may desire, and which can be made into tablets, will be manufactured to order. We believe this method of medication cannot fail to become more and more popular as its advantages grow to be appreciated.

